# Modeling Environmental Conditions in Poultry Production: Computational Fluid Dynamics Approach

**DOI:** 10.3390/ani14030501

**Published:** 2024-02-02

**Authors:** Erdem Küçüktopçu, Bilal Cemek, Halis Simsek

**Affiliations:** 1 Department of Agricultural Structures and Irrigation, Ondokuz Mayıs University, Samsun 55139, Türkiye; bcemek@omu.edu.tr; 2 Department of Agricultural and Biological Engineering, Purdue University, West Lafayette, IN 47907, USA; simsek@purdue.edu

**Keywords:** broiler, airflow, microclimate, simulation

## Abstract

**Simple Summary:**

The aim of this review is to provide researchers with a guide to simulating the environment of poultry houses using computational fluid dynamics. Through an extensive review of the literature in this area, it provides comprehensive insights into the common challenges encountered when applying this method, as well as a discussion of planned future research efforts.

**Abstract:**

In recent years, computational fluid dynamics (CFD) has become increasingly important and has proven to be an effective method for assessing environmental conditions in poultry houses. CFD offers simplicity, efficiency, and rapidity in assessing and optimizing poultry house environments, thereby fueling greater interest in its application. This article aims to facilitate researchers in their search for relevant CFD studies in poultry housing environmental conditions by providing an in-depth review of the latest advancements in this field. It has been found that CFD has been widely employed to study and analyze various aspects of poultry house ventilation and air quality under the following five main headings: inlet and fan configuration, ventilation system design, air temperature–humidity distribution, airflow distribution, and particle matter and gas emission. The most commonly used turbulence models in poultry buildings are the standard k-ε, renormalization group (RNG) k-ε, and realizable k-ε models. Additionally, this article presents key solutions with a summary and visualization of fundamental approaches employed in addressing path planning problems within the CFD process. Furthermore, potential challenges, such as data acquisition, validation, computational resource requirements, meshing, and the selection of a proper turbulence model, are discussed, and avenues for future research (the integration of machine learning, building information modeling, and feedback control systems with CFD) are explored.

## 1. Introduction

The global population, which stands at approximately 8 billion, is projected to reach nearly 10 billion by 2050 [1]. Meeting the food requirements of this expanding population requires a dual focus on increasing production capacities and improving production quality [2,3]. The ultimate objective is to achieve larger yields of superior-quality products per unit of agricultural land. This imperative has led to the widespread adoption of cutting-edge production techniques, including automation and mechanization systems [4,5,6].

With the application of these new technologies in livestock production, the global poultry market has experienced continuous growth, with production volumes rising from 97.32 million tons in 2019 to 102.04 million tons in 2022 [7]. This growth is primarily driven by the contributions of countries, such as the USA, Brazil, and China, with notable advancements also observed in Thailand, Mexico, and Türkiye [8]. It is further anticipated to increase by approximately 76% in 2050, reaching 180 million tons [9].

To ensure adequate food supplies for the growing population, it is critical to focus on modernizing poultry production. This includes advances in genetic breeding research and providing optimal environmental conditions for the birds. While much attention has been paid to breeding and feeding research in efforts to increase poultry production and productivity [10,11,12], it is important to acknowledge that the desired level of productivity cannot be achieved without appropriate environmental conditions in poultry houses [13]. Therefore, planning and designing poultry houses that provide optimal environmental conditions are imperative.

Effective planning and design of poultry houses involve a meticulous consideration of both the ideal indoor environmental conditions and the local climate data at the location [14]. The welfare and productivity of birds are closely intertwined with their immediate environment. It is, therefore, essential to create an environment that is conducive to their well-being and optimum performance. Failure to provide ideal conditions during the brooding phase can lead to reduced profitability, slower growth of birds, decreased feed intake, and increased disease susceptibility and mortality [15]. Historical data reveal the importance of providing an optimal housing climate. In the 1920s, the average mortality rate for broilers was about 20%. With advances in nutrition, house quality, and disease control methods, the mortality rate decreased by 4% by the end of the 20th century [16]. These experiences highlight the importance of providing an optimal indoor environment in poultry houses.

The assessment of environmental conditions within a poultry house can be significantly enhanced by applying modeling techniques. The models are specifically designed to simulate real-world scenarios using simplified approaches that deepen our comprehension of these situations and enable more accurate predictions for the future. In these models, heat and mass balance equations are solved to determine the factors, including animal species and age, building characteristics, and indoor and ambient conditions. These equations, which cannot be solved directly using analytical methods, are effectively tackled through numerical methods, such as computational fluid dynamics (CFD) [17,18].

Although numerous CFD studies have examined environmental conditions in various animal buildings, none have offered a comprehensive review that presents the current state-of-the-art research and development concerning environmental conditions in poultry buildings. Therefore, the primary objective of this review is to fill this gap by discussing the potential applications of CFD in evaluating poultry building environmental conditions. Additionally, it aims to address the existing challenges and recent advancements in physical models and numerical techniques within a typical commercial CFD workbench. 

This review emphasizes the critical importance of realism in CFD simulations and highlights the need for CFD modelers to address specific challenging issues. Furthermore, it explores various modeling techniques that can enhance the accuracy of CFD solutions for poultry building environments.

## 2. Importance of Environmental Assessment in Poultry Houses

**Figure 1:**
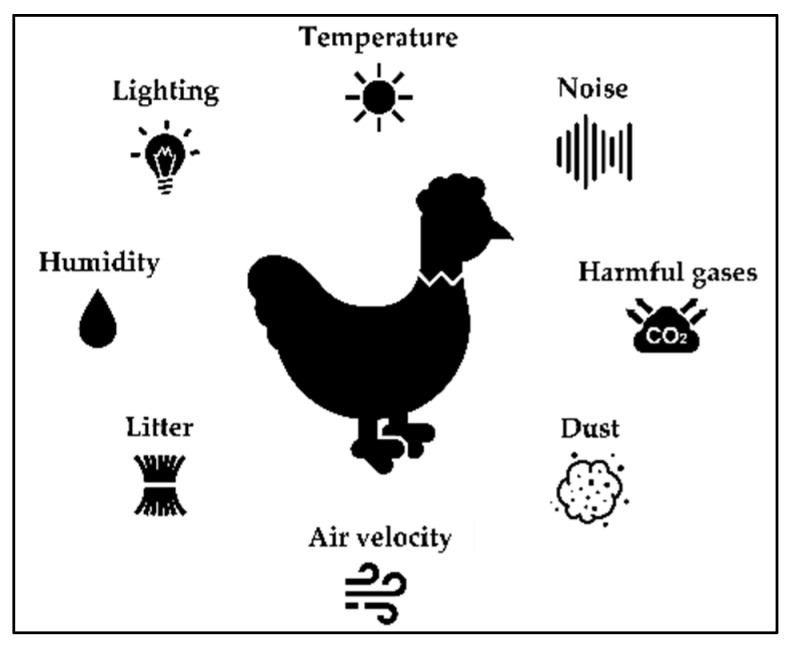
Environmental factors affecting the broilers’ performance.

Ensuring optimal air quality in poultry houses is crucial for the well-being of birds, with air temperature, humidity, velocity, and pollutant concentrations (Figure 1) being the key factors to consider [19]. To meet their requirements, a well-functioning ventilation system should maintain a continuous airflow, providing sufficient oxygen, appropriate humidity, velocity, and temperature while effectively removing gaseous pollutants. During summer, when external weather conditions are hot and humid, the temperature and humidity inside the poultry house tend to exceed the recommended ranges, leading to heat stress among the birds. This heat stress remains a prominent challenge in poultry farming. 

In a broiler house, it is recommended to initially set the indoor air temperatures between 30 and 33 °C and gradually decrease them to 24–26 °C over a period of 3–4 weeks. By the time 5–6 weeks have passed, the temperatures should be further reduced to a range of 18–21 °C [19,20]. 

High humidity levels in poultry houses can have detrimental effects on the well-being and growth of the birds [13]. To tackle this issue, it is crucial to implement adequate ventilation and temperature control strategies. During the summer season, increasing ventilation rates can effectively remove moisture and reduce humidity. During winter, raising indoor air temperature through heat sources can result in a reduction in relative humidity. For the first three days of a bird’s life, relative humidity of 65–70% is recommended, as humidity below 50% can lead to dehydration in chicks. After three days, indoor air humidity should range from 50% to 70%, considering the temperature and bird conditions [21,22]. 

Ensuring a consistent air velocity throughout the animal-occupied zone (AOZ) is crucial to prevent animals from migrating to areas with better ventilation. The concentration of birds in specific areas with improved airflow can lead to heightened mortality rates. Maintaining an air velocity of approximately 0.3 m s^−1^ during the first two weeks is generally recommended, increasing to 0.5 m s^−1^ in the third week, 1 m s^−1^ in the fourth week, and above 1 m s^−1^ afterward [23,24,25]. In hot weather conditions, when temperatures are elevated, a higher air velocity of approximately 2.5 m s^−1^ is necessary to provide adequate cooling for the chickens [26].

The presence of toxic gases, including carbon dioxide (CO_2_), ammonia (NH_3_), carbon monoxide (CO), and hydrogen sulfide (H_2_S), in poultry farms adversely affects bird performance and health of birds. Birds can tolerate pollutant gases up to certain concentrations, with the maximum allowable levels of 3000 ppm for CO_2_, 20 ppm for NH_3_, 10 ppm for CO, and 0.5 ppm for H_2_S [16].

## 3. Ventilation Systems for Poultry Houses

Effective ventilation is a cornerstone for successfully managing the environmental conditions within poultry houses [27]. Its paramount importance lies in its role as a key factor in alleviating respiratory problems in the birds, thereby promoting optimal productivity and achieving the highest conversion rates. 

Ventilation strategies for poultry houses range from fully natural ventilation (NV) to fully mechanical ventilation (MV) and hybrid natural–mechanical ventilation (NMV). With NV, the building design includes controlled side wall curtains and ridge vents. 

The NMV method was developed to replace the NV action specifically in cold weather, with the aim of improving control over the thermal environment. For warm and hot weather ventilation, the system relies on the opening of sidewall curtains and natural wind, while maintaining the same basic orientation requirements as a NV building. This strategic approach allows for greater adaptability to different weather conditions while ensuring efficient management of the indoor environment. The NMV method eliminates the need for cold weather ridge vents. Therefore, this method often utilizes a flat internal ceiling along with a heavily insulated attic for optimal thermal regulation [28]. 

In modern intensive poultry houses, an MV system with negative pressure is generally used. This system usually consists of exhaust fans connected in parallel, and fresh air is drawn in through the ceiling air outlets in cold to mild temperatures. In warmer to hot temperatures, on the other hand, the fresh air supply is facilitated by side wall and/or end wall curtains. The negative pressure MV system operates by creating a pressure differential inside the house, allowing air to flow from the inside to the outside. This configuration allows for efficient control of ventilation, temperature, and air quality [29]. 

## 4. Heating Systems for Poultry House

Poultry farms generally use two primary supplementary heating methods, radiant heaters and forced air space heaters, to ensure optimal environmental conditions for chick rearing [30,31]. The difference between these two methods lies in the way they deliver heat into the space.

Space heaters with forced ventilation heat the air in the building [31]. Although they have been used in the past, the recent trend in the poultry industry shows a move away from these heaters. The reason for this move is the observation that forced air heaters cannot effectively conduct heat to the floor, which is essential for warming the litter and thus maintaining an environment conducive to chick welfare.

In contrast, radiant heaters are an alternative that has gained popularity due to their effectiveness in directly heating the birds and the litter [32]. These heaters radiate heat energy and offer a more targeted and efficient way of warming the floor.

## 5. Fundamentals of CFD

**Figure 2:**
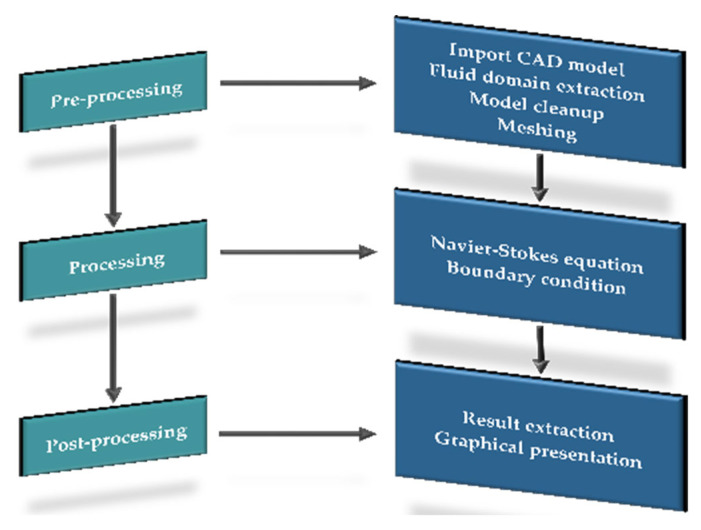
Computation procedure of a CFD simulation.

To conduct a CFD analysis, the analyst initiates the process by formulating the problem and employing scientific expertise to articulate it mathematically. Subsequently, the CFD software package incorporates this knowledge, translating the stated problem into scientific terms. Finally, the computer executes the calculations provided by the CFD software, and the analyst reviews and interprets the results [33]. The CFD analysis consists of three main steps: pre-processing, processing, and post-processing (Figure 2).

### 5.1. Pre-Processing

**Figure 3:**
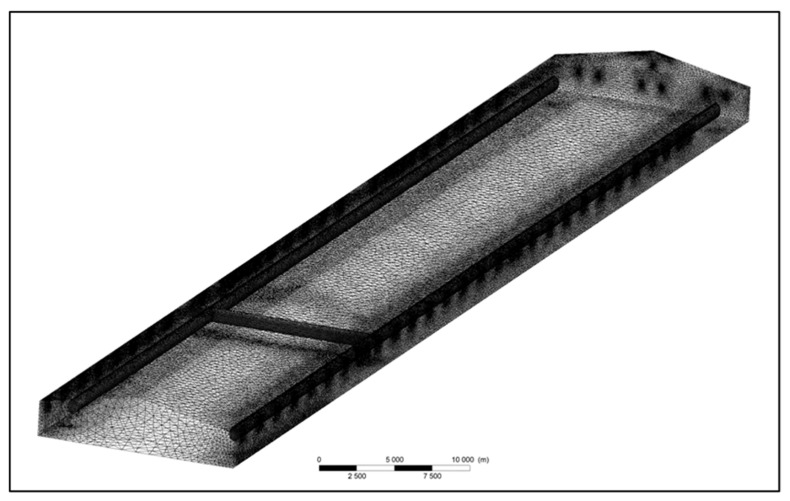
Meshing structure of a commercial broiler house [34].

The simulation relies on thorough problem analysis, which is critical to defining precise goals and parameters and fully understanding the underlying problems. Defining the physics of the problem forms the foundation, followed by creating a problem-specific two- or three-dimensional geometry for accurate problem analysis. The final step of pre-processing is then discretization or meshing. Figure 3 shows an example, which is the meshing structure of a commercial broiler house [34]. The continuous fluid domain is subdivided into smaller, discrete control volumes or cells. This subdivision can be performed using a variety of techniques, such as structured grids or unstructured meshes. The choice of meshing technique depends on the nature of the problem and the computational resources available. Once the meshing is complete, the boundaries of the problem domain can be determined, and the required boundary conditions defined in the initial phase can be applied.

### 5.2. Processing

Processing involves using a computer to solve the mathematical equations of fluid flow. Once the domain is discretized, the analysis is performed for each control volume. The governing equations, such as the Navier–Stokes equations for fluid flow, are applied to each control volume to establish a system of equations. These equations describe the conservation of mass (Equation (1)), momentum (Equation (2)), and energy (Equation (3)) in each volume. 

Conservation of mass (continuity equation): The net mass flow into or out of a control volume must be balanced.

(1) $$\frac{\partial \rho }{\partial t}+\frac{\partial }{\partial x_{j}}\left(\rho u_{j}\right)=0$$

Conservation of momentum (momentum equation): The sum of external forces acting on a fluid particle equals the rate of change in its linear momentum.

(2) $$\frac{\partial }{\partial t}\left(\rho u_{j}\right)+\frac{\partial }{\partial x_{j}}\left(\rho u_{i}u_{j}\right)=\frac{\partial }{\partial x_{j}}\left[-p\delta_{ij}+\mu \left(\frac{\partial u_{i}}{\partial x_{j}}+\frac{\partial u_{j}}{\partial x_{i}}\right)\right]+\rho g_{i}$$

Conservation of energy (energy equation): The rate of change in the energy of a fluid particle equals the sum of heat addition and work performed on the particle.

(3) $$\frac{\partial }{\partial t}\left(\rho C_{a}T\right)+\frac{\partial }{\partial x_{j}}\left(\rho u_{j}C_{a}T\right)-\frac{\partial }{\partial x_{j}}\left(\lambda \frac{\partial T}{\partial x_{j}}\right)=S_{T}$$

where *ρ*: density (kg m^−3^), *t*: time (s), *x*: Cartesian coordinates (m), *u*: velocity component (m s^−1^), *p*: pressure (Pa), *δ*: Kronecker delta, *μ*: dynamic viscosity (kg m^−1^ s^−1^), *g*: acceleration due to gravity (m s^−2^), *C_a_*: specific heat capacity (W kg^−1^ K^−1^), *T*: temperature (K), *λ*: thermal conductivity (W m^−1^ K^−1^), *S_T_*: thermal sink or source (W m^−3^), and *I* and *j*: the Cartesian coordinate index. 

In this step, it is necessary to refer to some turbulence models. A bewildering variety of such models can be found in the literature, the two main classes being Reynolds-averaged Navier–Stokes models (RANS) and large eddy simulation (LES). The choice of turbulence model depends on the specific flow problem, the available computational resources, and the desired level of accuracy [35]. Engineers select an appropriate turbulence model based on the flow characteristics, Reynolds number, boundary conditions, and analysis objectives. After determining the appropriate turbulence model, numerical methods are used to iteratively solve the equations, often using finite difference, finite volume, or finite element methods. These methods provide solutions at discrete points within each control volume, known as nodes. These solutions provide valuable information about the fluid variables at these locations, such as velocity, pressure, temperature, and species concentration. 

### 5.3. Post-Processing

**Figure 4:**
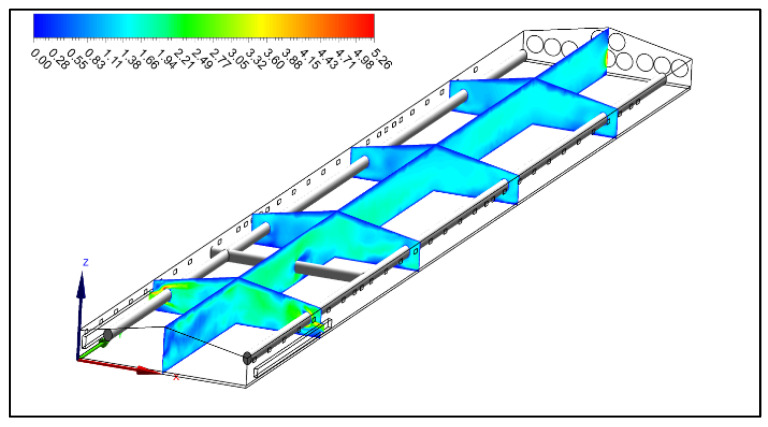
Airspeed contours in a commercial broiler house during summer [36].

Once the simulation phase produces results, the next step is to analyze them. Various methods can be used for this analysis, including vector plots, contour plots, data curves, and streamlines. These graphical representations often employ colors to distinguish between different value ranges. For example, Figure 4 illustrates airspeed (m s^−1^) contours in different planes within a commercial broiler house during the summer [36]. These visualizations help grasp the distribution of airspeed within the enclosed environment.

## 6. Advantages and Limitations of Using CFD

CFD offers a dual perspective with notable advantages and inherent limitations. On the positive side, it serves as a cost-efficient and time-saving alternative to traditional experimental methods. It allows the exploration of different scenarios and design iterations in a virtual environment. The ability of CFD to provide detailed insights into fluid dynamics, pressure, and temperature in simulated areas provides engineers with valuable data for design optimization. It also enables analysis of the hard-to-reach regions, helping to identify problems early in the design phase [37]. However, there are limitations, mainly related to the accuracy of the mathematical models and the dependency on the mesh [38]. The validity of CFD results depends on accurate models, and the dependence on the quality and size of the mesh can be challenging. Complex simulations often require significant computing resources, which limits access to high-performance computers. In addition, user expertise is critical, as incorrect applications and simplifying assumptions can lead to inaccurate conclusions. Despite these limitations, using CFD with experimental validation remains a practical approach for understanding and optimizing fluid dynamics in various engineering applications.

## 7. Role of CFD in Environmental Assessment

**Figure 5:**
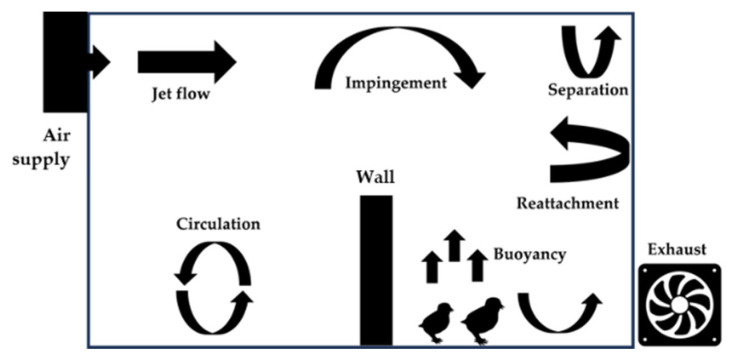
Typical flow characteristics within an enclosed environment with diverse flow mechanisms.

Air distribution in an enclosed environment can be influenced by different forces, including natural wind, mechanical fans, and thermal buoyancy. The complex airflow patterns, including circulation, reattachment, separation, vortex impingement, and buoyancy effects, are a result of these forces interacting with each other and with the geometry of the enclosed space, as illustrated in Figure 5 [39].

The flow regime within an enclosed environment can range from laminar to transitional to turbulent flows and sometimes a combination of these regimes, especially under transient conditions. The complicated nature of indoor air flow poses a significant challenge for experimental studies, as they are usually cumbersome and costly. However, with the rapid advancements in computer capacity and speed, CFD has emerged as a powerful alternative for predicting airflows in enclosed environments [38]. By solving the conservation equations for mass, momentum, energy, and species concentrations, CFD can provide quantitative calculations for various air distribution parameters within an enclosed space.

## 8. Commercial Software and Open-Source CFD Codes

Advancements in computer hardware, numerical methods, and software development have contributed to the enhanced capabilities of CFD codes. Numerous commercial software and open-source CFD codes are available, each with their strengths and specializations. For example, Ansys-Fluent, with its user-friendly interface and robust pre- and post-processing capabilities, is ideal for complex industrial simulations. It offers a comprehensive library of turbulence models and multiphysics functions. In the literature, many researchers have successfully employed this software to model environmental conditions in poultry houses [40,41,42,43]. Its excellent design capabilities make it suitable for solving comprehensive interdisciplinary problems. Star-CCM+ is more efficient and convenient than general CFD software because it performs result processing without separate post-processing [44]. Phoenics proves valuable in process engineering simulations, especially in modeling fluid flow and heat transfer in applications, such as airflow in buildings and HVAC systems [45,46,47], allowing steady-state and transient simulations. Acusolve efficiently handles large and complex simulations with parallel processing capabilities and various turbulence models to simulate turbulent flows accurately [48,49]. PAM-Flow, known for its robust capabilities, is ideal for simulating external aerodynamics, under-hood flows, and thermal management of vehicles [50]. The main commercial CFD software is listed in Table 1. In addition, OpenFOAM, SU2, Gerris Flow Solver, Code_Saturne, and Elmer are the most widely used open-source CFD codes for the simulation of flows and related phenomena.

## 9. Case Studies and Applications

CFD is a powerful simulation tool that uses computers and applied mathematics to model flow situations, heat, mass, and momentum transfer. Its applications in optimizing the design and performance of agricultural buildings are precious. However, the scope of this review is limited to studies explicitly examining the use of CFD to determine the microclimate in poultry houses. The indoor climate in poultry houses is critical to animal well-being and productivity. Air temperature, relative humidity, pollutant concentration, and air movement significantly impact the birds’ ability to regulate their body temperature and maintain homoiotherm. Therefore, understanding and controlling these parameters are critical to creating optimal conditions for poultry production [16].

CFD can be applied to study and analyze various aspects of poultry house ventilation and air quality under the following five main headings: inlet and fan configuration, ventilation system design, air temperature–humidity distribution, airflow distribution, and particle matter and gas emission.

### 9.1. Inlet and Fan Configuration

Improving climate control for poultry houses depends primarily on accurately controlling the ventilation rates throughout the facility. Although suitable equipment is available, its use in most houses is limited. Another significant challenge is the uneven distribution of airflow within the building. The airflow pattern within the poultry house serves as the crucial connection between the incoming air and the microenvironment surrounding the birds. Therefore, accurately controlling the airflow pattern is a priority [51].

The airflow patterns within a poultry house are greatly influenced by the building’s geometry and the positioning of fans and inlets [52,53,54]. Using CFD, Cheng et al. [26] explored the effect of inlet position and flap on airflow and temperature in a laying hen house. The ventilation volume employed in the simulation corresponded to that observed during the field trial, amounting to 229 m^3^ s^−1^. The boundary conditions replicated those of the validated model, with the exception of the AOZ, which was simplified using the porous media approach. Accordingly, the heat generation rate within the AOZ was set at 456.67 W m^−3^. The findings revealed that the positioning of the inlet and the presence of a flap had a significant impact on airflow and temperature near the inlet. Increasing the inlet area in the gable wall and establishing a greater distance between the cages and sidewall inlets led to higher airspeed, lower temperatures, and improved airflow distribution. However, the study does have limitations. For instance, it did not explore how humidity in the laying hen house varies with different inlet positions. Additionally, the thickness of the evaporative pad might impact inlet air velocity, and the angles of the flaps could change with varying inlet air velocity, affecting the reach of the inlet airflow to the center of the ceiling. It is crucial to note that this study exclusively focused on laying hen houses, and its findings may not be directly applicable to other poultry housing systems.

Tong et al. [55] conducted a study investigating the influence of different opening percentages of air inlets on the airflow pattern. In their CFD study, the air inlets were simulated as pressure inlets, where the incoming air was determined by the static differential pressure between the interior and the atmosphere. Considering that air inlets can be variably opened from 0% to 100% in practical scenarios, the inlet structure within the computational domain was configured to include different opening percentages, namely, 25%, 37.5%, 50%, 62.5%, 75%, and 100%. The researchers noted that the air velocity distributions exhibited reduced magnitudes and less variability during autumn and winter compared to the summer scenario. These differences were linked to decreased house ventilation rates and the partially opened baffle inlets. Specifically, during autumn and winter, the baffle inlets were opened at smaller angles, forming vortex airflow patterns due to high-velocity air streams near the inlet.

In a study investigating the effects of fresh air vents on poultry house aerodynamics, Trokhaniak et al. [56] proposed several recommendations. Firstly, they suggested the installation of spoilers positioned above the fresh air valves at an angle of 75° from the vertical line. Additionally, they recommended that the exterior walls be attached to the inside of a concrete frame. Furthermore, they proposed expanding the width of the poultry house to a maximum of 22.36 m. Finally, they advised reducing the height of the flooring to approximately 3.9 m above the floor level.

Du et al. [57] conducted a study to optimize the air inlet configurations using the CFD model. For this study, a 3D CFD model was constructed to replicate the real dimensions of a laying hen house, and its accuracy was validated by comparing the simulation results with field measurements at specific positions. Given the inherent complexity of numerical simulation of a 3D real-world scenario, certain approximations were unavoidable. Recognizing that it is impractical to model each bird within the geometric representation, the researchers employed a porous media model to simulate the AOZ, ignoring specific details related to the feeding and water supply systems. The CFD results revealed that the positioning of all the air inlets on the side walls of the building resulted in significant vorticity at the front, suggesting uneven air movement within the facility. However, when the air inlets were relocated to the front wall, a more uniform flow pattern with reduced vorticity was achieved. In addition, the researchers used CFD simulations to investigate the effects of correctly sized air inlets installed in the center of the side walls. They found that this configuration could effectively decrease the expected high temperatures at the end of the building without the need to increase the ventilation rate or consume additional energy.

Kucuktopcu and Cemek [58] applied the CFD technique to enhance their comprehension of indoor conditions in poultry houses, offering valuable insights for producers and end-users to improve management decisions. The simulation results made it clear that there are stagnant areas opposite the fans within a broiler house. To address this issue, they recommended a corrective measure of shutting off inlets closer to the fan region and opening inlets further away from the fans. By doing so, they explained that the high static pressure created in this region would facilitate better air mixing and alleviate the presence of stagnant areas. This adjustment was proposed to enhance the air circulation and create more favorable conditions for the poultry house environment.

Zou et al. [59] examined the impact of installing windshields on the airflow distribution in poultry houses during summer. By analyzing CFD simulation results, the researchers determined the optimal installation height for the windshield. The findings revealed that applying windshields led to a notable increase in wind speed and reduced temperature inside the house. The numerical simulations also showed that the appropriate installation height should be 1.8 m.

### 9.2. Ventilation System Design

Ventilation systems are essential in poultry production to control environmental conditions, and their design is also of paramount importance, as the airflow patterns in the house determine the characteristics and uniformity of environmental parameters.

Guerra Galdo et al. [60] conducted a CFD simulation study to analyze and compare three poultry house designs: tunnel, semi-tunnel, and improved semi-tunnel. These designs featured different configurations of inlets and fans. The study implemented a 2D steady-state model assuming constant density and second-order flow. The model considered the production of sensible heat, which was estimated for 5-week-old animals with a body weight of 2.5 kg. Heat production was included in the model as a uniform flux of sensible heat (101.94 W m^−2^) from the concrete floor. The CFD simulation results revealed that the improved semi-tunnel configuration performed better than the tunnel and semi-tunnel designs.

Seo et al. [61] developed and modeled four modified ventilation systems (a chimney, a side vent, a pipe under the roof, and a side-up vent at the eaves) for a naturally ventilated broiler house. The CFD models were created based on heat production of 110.5 W m^−2^, considering the entire floor area occupied by broilers. The outside air temperature was set at 0.6 °C, while the inside was assumed to be 25 °C. The CFD simulation results indicated that, unlike the conventionally controlled model, the upgraded model was governed by an on–off timer responsive to the inside temperature, reducing dependence on arbitrary atmospheric conditions. Furthermore, among the configurations tested, it was observed that the model with a diffuser under the chimney inlet exhibited the best performance.

The tunnel ventilation system is widely used to control the indoor environment in poultry houses. However, maintaining an optimum indoor temperature in winter proves to be challenging. This difficulty in regulating the temperature can lead to cold stress in the chickens and affect their production performance. To solve this problem, Yang et al. [62] introduced an innovative double-duct ventilation system that combines the benefits of an exhaust air heat recovery system and a perforated duct ventilation system using CFD techniques. The novel double-duct ventilation system comprises multiple units evenly distributed along the side walls, each of which can be controlled independently for precise environmental management. These ventilation devices consist of an inner duct, outer duct, fresh air fan, and exhaust air fan. The total ventilation rate for the novel double-duct system was set at 65,300 m^3^, which is consistent with the tunnel ventilation system installed in the poultry building. For the numerical analysis, the complexity was reduced by simplifying each tier of cages to one resistance unit. To simulate the resistance effect of the chickens and cages on the airflow, the loss coefficients along three directions were set as 1.2 m^−1^, and the porosity was set as 0.85 in the resistance unit. The heat production of the chickens was assumed to be 4.1 W kg^−1^. The authors emphasized that the innovative double-duct ventilation system proves to be extremely effective in preheating cold fresh air, preventing cold stress in chickens, and creating a favorable thermal environment. Compared to the conventional tunnel ventilation system, the novel double-duct ventilation system resulted in a significant increase in the average indoor temperature of the poultry house, with a significant increase of 4.4 °C.

In a study conducted by Babadi et al. [63], the authors focused on developing an effective ventilation system by addressing the limitations identified in the models proposed by Wang and Wang [64]. Three new models were introduced, each of which was simulated by adjusting the dimensions of the evaporative cooling pads and changing the number and position of the exhaust fans. The evaluation showed that the second new model, with fifteen fans on the east wall and three evaporative cooling pads on the west wall, performed the best.

Tong et al. [42] introduced the upward airflow displacement ventilation (UADV) system, a novel ventilation system. To assess the performance of the UADV system, the researchers compared it with a typical tunnel ventilation (TV) system using the CFD technique. The study findings demonstrated that the UADV system exhibited 46–129% higher air exchange effectiveness within the cages than the TV system. This study contributes to our comprehension of ventilation system design, yet additional research is crucial for the practical implementation of the UADV system in commercial layer houses. Further studies are required to delve into specific design considerations for air inlet ducts and the ceiling, ensuring practical and effective application in real-world production environments.

Another study by Bustamante et al. [65] utilized CFD to investigate the efficiency of ventilation systems and identify optimal designs for poultry houses. Their findings revealed that mechanical cross-ventilation systems, although suitable for common weather conditions, may not be sufficient in preventing mortality episodes caused by heat stress. This is attributed to the fact that these systems provide lower air velocity values than what animals require under such challenging conditions. The authors emphasized the importance of future instrumentation efforts and specifically advocated the development of multi-sensor systems capable of providing isotemporal measurements of air velocity components. They also stressed the importance of considering animal thermal comfort in research focused on characterizing buildings and identifying key elements for optimal poultry farms.

### 9.3. Airflow Distribution

Poultry houses with inadequate ventilation systems are prone to high mortality rates, especially when the microenvironment around the birds becomes hot, humid, and stagnant. It is crucial to maintain uniform air velocity within the bird-occupied zone to deter their migration toward better-ventilated but already crowded areas, as this behavior can ultimately result in a higher mortality rate among the birds.

Cheng et al. [66] conducted a study to address the issue of reduced air speed in the AOZ during summer tunnel ventilation in a hen house. Typically, a large free space beneath the ceiling is designed to fully mix the cold inlet air with room air during winter. However, this design is not optimal for tunnel ventilation, as a significant portion of the ventilation air passes through the free space under the ceiling instead of the AOZ. To overcome this problem, the researchers investigated the application of deflectors beneath the ceiling CFD simulations. Deflectors were introduced to redirect the airflow toward the AOZ, thereby increasing air speed and the wind chill effect. The study analyzed the effects of different heights and intervals of the deflectors along the length direction of the cage. The findings revealed that the variation trends of airspeed were nearly identical under different heights of deflectors, indicating that height variation had minimal impact on airflow distribution. However, significant differences in airspeed variation trends were observed under varied intervals of the deflectors. This suggested that the spacing between the deflectors played a crucial role in determining the airflow distribution and air speed within the AOZ. The study also found that the uniformity of air speed distribution was positively related to the height of the deflectors and negatively related to the interval between them. This means that higher deflectors and closer spacing between them contributed to a more uniform air speed distribution within the AOZ.

In a study conducted by Blanes-Vidal et al. [67], a comprehensive assessment of ventilation system designs was performed to evaluate the effectiveness of these designs in generating suitable air velocities within the AOZ. The authors performed four CFD simulations to analyze the airflows in a mechanically ventilated commercial poultry house. The accuracy of these simulations was verified by comparing the simulated and measured air velocities. In scenario 1, the inlet velocity was assumed to be uniformly distributed across all inlet areas. In scenario 2, the air velocity at each inlet was equated to the time-averaged mean velocity measured at each specific inlet using a portable hot wire anemometer. In scenario 3, the atmospheric pressure was used as the boundary condition at the inlets, while the air velocity was fixed at the outlets. Finally, in scenario 4, the boundary conditions ‘velocity at the inlets’ and ‘negative relative pressure at the outlets’ were used. The findings indicated that refining CFD results by including at least 15 indoor air velocity measurements led to a more accurate estimate of the mean air velocity at the height of the birds. The authors, therefore, concluded that the study could provide valuable insights into the actual airflow dynamics in commercial poultry houses. However, they also pointed out the practical challenges encountered in measuring and modeling real poultry houses during the study.

Chen et al. [41] employed CFD modeling techniques to quantify the efficiency of ventilation systems in maintaining optimal and consistent living conditions, particularly at the level of individual hens. A noteworthy aspect of the study was the introduction of a comprehensive CFD model that included individual hen models. This approach significantly enhanced the robustness of their assessment of the birds’ welfare conditions. However, the authors pointed out that various factors, including the relative location of inlets and exhaust fans and the dimensions of baffles, play a crucial role in shaping the indoor environment. Therefore, they suggested that further CFD analysis could investigate the uniformity of temperature and air velocities together with environmental parameters at the individual hen level to address practical issues, such as floor eggs.

### 9.4. Air Temperature–Humidity Distribution

Hot and humid conditions in poultry houses adversely affect bird welfare and productivity [52,68]. As temperatures and humidity rise, heat stress becomes a problem, resulting in reduced feed intake, lower meat/egg production, and increased mortality [69]. To overcome these challenges, it is critical to implement sustainable ventilation designs that can maintain an optimal thermal environment for poultry. However, relying only on maximum ventilation may not be sufficient in certain hot weather conditions, requiring evaporative fan–pad cooling systems [70,71]. During the summer, the prevailing ventilation practice involves operating an evaporative cooling system and installing cooling pads on the gable wall and/or both sidewalls at one end of the building while fans are placed at the opposite end [72].

The CFD study by Küçüktopcu et al. [36] demonstrated the potential benefits of implementing evaporative fan–pad cooling systems in summer. Implementing an evaporative cooling pad system during the summer effectively mitigated high temperatures. When the mean air temperature inside the house exceeded 25 °C, this system resulted in an approximate decrease of 3 °C. Notably, in areas near the pads where air temperatures were low (22.16–22.88 °C), there was a gradual increase of almost 2.50 °C from the pads to the exhaust fans.

In other studies, Al Assaad et al. [73] conducted a comparative analysis of the effectiveness of three passive cooling systems in meeting thermal comfort and indoor air quality standards within a poultry house situated in a semiarid climate. The first two systems under investigation were a direct evaporative cooler and a crossflow dew point evaporative cooler. These systems deliver air through conventional tunnel ventilation to ensure uniform thermal conditions and indoor air quality. The third system introduced a dewpoint evaporative cooler and paired it with a localized ventilation system, aiming to achieve additional reductions in air and water consumption while maintaining desired environmental conditions in the poultry house. The research findings revealed that the dewpoint evaporative cooler performed better than the direct evaporative cooling system.

Wang and Wang [64] employed CFD techniques to comprehensively analyze the environmental conditions in a mechanically ventilated commercial layer house equipped with a wet pad cooling system. In the simulation of the original house, two improved cases were created to improve the indoor environmental conditions. In improved case 1, two pads originally installed in the side walls were removed. In improved case 2, five exhaust fans were installed on each end wall with a distance of 1.9 m between the fans, and two wet pads were placed in the center of each side wall. The simulations showed that of the three cases, improved case 2 proved to be the optimal choice, as it created an environment with reduced opportunities for layers to experience severe heat stress conditions, attributed to the younger age of the air. The main limitation of this study is that it does not take into account relative humidity, which is a crucial factor for the performance and production of laying hens.

The distribution of environmental variables and thermal comfort provided a more comprehensive understanding of ventilation and thermal conditions in the poultry house and provided insights to improve facility design and management to enhance poultry welfare [74,75,76]. In a specific study focused on thermal comfort issues, Tong et al. [55] employed a 3D CFD model to assess the temperature–humidity index (THI) and heat stress levels during summer, autumn, and winter. The study findings revealed that heat stress was prevalent in 69.1%, 78.0%, and 18.4% of cages during summer, autumn, and winter, respectively, as determined by the THI. Furthermore, 18.3% of the cages experienced cold stress during winter due to low incoming air temperature and insufficient air mixing.

### 9.5. Particle Matter and Gas Emission

Particulate matter (PM) and gas emissions constitute a significant air pollution concern for commercial poultry production facilities [77].

Knight et al. [78] developed a comprehensive COMSOL software model to simulate the poultry PM collection process in an electrostatic precipitator (ESP) module. The model offered a cost-effective alternative to physical prototypes for assessing ESP designs aimed at mitigating PM in poultry facilities. Pawar et al. [79] investigated the PM and gaseous contaminant levels in laying hen houses under different ventilation scenarios.

Some studies have analyzed the spatial distribution of NH_3_ concentration in poultry houses [80,81]. Gonçalves et al. [17] developed a numerical model to accurately forecast the velocity fields within the domain and estimate the dispersion of NH_3_ emissions from the poultry litter in the housing. Six different situations were simulated to replicate summer, winter, and mid-season periods, including the day and night periods. The findings revealed that in winter and summer configurations, characterized by lower extraction flow rates, there was a considerable increase in NH_3_ concentration along the tunnel, with inefficient removal of NH_3_. Conversely, in situations with higher air extraction flow rates (mid-season), NH_3_ was effectively removed, leading to consistently low concentrations throughout the tunnel.

## 10. Practical Challenges in Implementing CFD in Poultry Production

CFD is a valuable tool for analyzing airflow and ventilation in poultry buildings. It also provides insights into the distribution of pollutants, thermal conditions, and potential areas of improvement for bird health and performance. However, it does have certain limitations and challenges. In this section, we present some limitations and solutions on the application of CFD, which should be addressed in future research.

### 10.1. Data Acquisition and Validation

Data acquisition involves collecting relevant information to monitor and control the poultry production environment. In the context of CFD, data acquisition primarily focuses on gathering data related to environmental parameters, including temperature, humidity, air velocity, gas concentrations (e.g., CO_2_, NH_3_), and particulate matter levels in the poultry house.

To achieve effective data acquisition in poultry production, sensor systems must be strategically placed throughout the facility [36]. These sensors continuously gather real-time data, which is then transmitted to a central control system or cloud-based platform. Advanced sensor technology and Internet of Things (IoT) devices have made collecting and managing this data easier, providing poultry farmers with a comprehensive view of their operation [82].

Validation is critical in ensuring simulated CFD models accurately reflect real-world poultry production conditions. A CFD model that is not compared with measurements would not be credible and, consequently, could not be used as a relevant tool to test the system’s response under different operating conditions [83]. Validation involves comparing CFD predictions with actual measurements collected by sensors in the poultry house [65,67]. Nevertheless, obtaining accurate and practical results for fluid dynamic factors through field experiments is inherently challenging. This challenge arises from the inherent variability and instability of field conditions, which require significant investment in time and labor. Therefore, some research teams have explored alternative approaches to optimize their experimental conditions. These approaches include wind tunnel tests, Particle Image Velocimetry (PIV) tests, scale model simulations, and similar methods [84,85]. Additionally, some researchers have turned to pre-existing data generated by previous investigators with similar research objectives to validate their CFD models [63].

### 10.2. Computational Resource Requirements

Simulations can be computationally demanding and require significant computational resources and time [86,87]. Handling large-scale simulations for entire poultry buildings with multiple scenarios can pose computational power and storage capacity challenges. The computational demands of CFD simulations arise from the need to solve complex equations that describe the behavior of fluids and their interactions with solid surfaces. These simulations involve dividing the domain into a grid or mesh of small computational cells and solving the governing equations for each cell. As the domain size and the simulations’ complexity increase, the number of cells and the computational time required also increase. However, advancements in computing technologies and optimization techniques continue to improve the efficiency and accessibility of CFD simulations.

### 10.3. Simplification and Validity of Assumptions

In small-scale animal barns with relatively few animals, it is feasible to include detailed models of individual animals in CFD simulations [88]. However, for larger barns and a higher number of animals, the computational requirements become more demanding, and the inclusion of detailed animal models becomes impractical. Therefore, the presence of animals has often been neglected in several studies, as large-scale simulations with high mesh numbers present computational challenges [65,67]. The limited computational power of ordinary desktops or professional workstation computers may not be sufficient to handle such simulations [89].

To simplify the geometric modeling of the AOZ, researchers have turned to porous media models in CFD simulations of barns. These models represent animal blocks and/or slatted floors as porous regions, allowing for more computationally intensive simulations [90,91]. Porous media models provide an effective trade-off between computational efficiency and capturing the effects of animals on airflow and ventilation within the barn.

Applying the porous media assumption in CFD simulations of a poultry house simplifies the complicated geometry of the AOZ to a porous region. The idea of porous media is to insert a source term in Navier–Stokes equations. The source term consists of two parts: the viscous loss term (Darcy’s law) and the inertial loss term (Equation (4)).

(4) $$\frac{\Delta P_{i}}{\Delta \chi_{i}}=-\left(\sum_{j=1}^{3}D_{ij}\mu \nu_{j}+\sum_{j=1}^{3}C_{ij}\frac{1}{2}\rho \left|\nu \right|\nu_{j}\right)$$

where $∆P_{i}/∆\chi_{i}$: the pressure drop per unit length for the *i*th (x, y, or z) direction (Pa m^−1^), *μ*: the dynamic viscosity of air (Ns m^−1^), *ν_j_*: the inlet air velocity in x, y, or z directions (m s^−1^), *D*: the matrix of viscous resistance coefficients (m^−2^), *C*: the matrix of inertial resistance coefficients (m^−1^), *ρ*: the air density (kg m^−3^), and ∣*ν*∣: the magnitude of the velocity (m s^−1^).

Resistance coefficients are employed to capture the relations between inlet velocities and flow resistance within the AOZ. These coefficients are usually determined by empirical correlations or experimental measurements. Performing CFD calculations to evaluate pressure drops across the AOZ involving discretely modeled birds and varying inlet velocities provides a practical way to derive the resistance coefficients. This approach allows for a simulation of the flow behavior and pressure drops within the bird zone under various operating conditions [92].

Some studies have confirmed the feasibility of applying porous media in poultry houses in some cases. For example, Du et al. [57] demonstrated the successful application of porous media models in studying airflow distributions, temperature, and relative humidity in a poultry house. By simplifying the AOZ as a porous media, the researchers could simulate the airflow patterns and environmental conditions within the poultry house. Cheng et al. [92] examined how the geometry, arrangement, and weight of birds inside cages impacted the flow resistance experienced by laying hens. They calculated resistance coefficients by modeling the area around the birds as a porous medium in various scenarios using CFD techniques. The simulation results revealed a significant impact of hen distribution on flow resistance. The variation in flow resistance was attributed to the positive correlation of the drag force with the projected area. Different distributions could significantly influence the projected area perpendicular to the flow directions. This alteration in the projected area could lead to increased drag force and thus increased momentum loss. Consequently, air velocity and flow resistance in the AOZ could be affected. Also, body weight had a significant effect on flow resistance, and flow resistance had a lesser variation with increased body weight. 

### 10.4. Selection of a Proper Turbulence Model

Selecting an appropriate turbulence model is crucial in developing CFD simulations. Turbulence models are necessary to provide closure for the Navier–Stokes equations, which describe fluid flow behavior. The challenge with turbulence modeling is that no single model can accurately represent all Reynolds flow regimes. Reynolds flow regimes refer to the range of flow conditions characterized by the Reynolds number, which determines the relative importance of inertial forces to viscous forces in the flow. Different turbulence models are designed to capture specific turbulence characteristics under certain flow conditions [39,93].

The choice of a turbulence model depends on several factors, including the specific flow conditions, available computational resources, and desired accuracy. It often involves a trade-off between accuracy and computational cost. It is common practice to validate turbulence models against experimental data or higher-fidelity simulations to assess their performance for a particular application.

In the field of CFD, numerous turbulence models have been proposed in the literature. Choosing an appropriate model is crucial for predicting the indoor environment of poultry buildings. A review of CFD applications in poultry buildings revealed that the most commonly used turbulence models were the standard k-ε, renormalization group (RNG) k-ε, and realizable k-ε models (Table 2).

Unfortunately, a lack of comprehensive research has thoroughly examined the impact of different turbulence models on the accuracy of predictions regarding the indoor environment of poultry houses. To address this gap, Kucuktopcu and Cemek [40] focused on evaluating the performance of three different variants of the k-ε turbulence model: the standard k-ε, RNG k-ε, and realizable k-ε. The objective was to determine which model provided the most accurate simulation of the internal turbulent flow in a poultry house. Their findings indicated that the RNG k-ε turbulence model agreed best with the measurements of airspeed and temperature. Therefore, its use and typical parameters were recommended for simulating the indoor environment of poultry houses.

## 11. Future Research Directions

### 11.1. Hybrid CFD and Machine Learning (ML) Approaches

Hybrid approaches combining CFD and ML techniques have gained significant attention recently, especially in fluid dynamics and engineering [94,95]. These approaches leverage the strengths of both CFD and ML to enhance the accuracy, efficiency, and robustness of simulations and extract valuable insights from complex fluid flow problems [96,97].

ML algorithms can be used to generate training data for CFD simulations. This can help reduce the computational cost of running CFD simulations, especially for problems with high-dimensional parameter spaces. Also, ML algorithms can improve turbulence modeling in CFD simulations. They can learn from experimental data or high-fidelity simulations to better capture complex turbulent flows, reducing the reliance on traditional turbulence models. The hybrid method has proven valuable in tackling the computational challenges associated with CFD models for fluid flow. This method has yielded promising results, effectively balancing accuracy with computational efficiency [98,99].

### 11.2. Coupling CFD with Building Information Modeling (BIM)

Coupling CFD with BIM is a forward-looking approach that can enhance buildings’ design, energy efficiency, and overall performance [100]. BIM-CFD integration facilitates early-stage analysis during the design phase and optimizes airflow and temperature distribution [101]. This can lead to energy savings, reduced HVAC system sizes, and improved thermal comfort. As technology continues to advance, this integration is likely to play an increasingly essential role in the construction and operation of buildings in the future.

### 11.3. Real-Time CFD Simulations and Feedback Control Systems

Real-time CFD simulations and feedback control systems can be integrated to create closed-loop control systems. In such systems, CFD simulations provide real-time data about the fluid flow, which are then used as feedback to adjust control parameters in an automated or semi-automated manner. This integration offers significant advantages in process optimization, efficiency improvement, and safety in a wide range of industries [102,103,104,105]. These technologies continue to evolve as computational capabilities and control strategies advance.

## 12. Conclusions

This paper comprehensively reviews the latest advancements in CFD modeling techniques for assessing environmental conditions in poultry houses and extensively covers the practical challenges associated with implementing CFD in different poultry production settings. From this review paper, the following conclusions can be made.

Careful validation of CFD results is crucial to ensure their accuracy and predictive capability. The integration of CFD simulations with experimental research provides a comprehensive insight into the climatic conditions in poultry houses. The use of CFD simulations can be particularly valuable in the initial stages of poultry house design, as it allows for the exploration of potential alternatives and design improvements.

Modeling the exact position and size of the birds is essential to achieve the most accurate solutions. This approach is particularly important as the interior of the house is inhomogeneous and there is an inherent resistance to airflow at the level of the birds. In cases with complex geometries and poultry houses with a higher number of birds, it is advisable to simplify the geometric modeling of the AOZ using a porous media model and introduce a uniform heat flux into the CFD model.

The overview of CFD applications in poultry houses shows that the most commonly used turbulence models are the standard k-ε, RNG k-ε, and realizable k-ε models. However, it is noted that there is a lack of comprehensive research that thoroughly investigates the effects of the different turbulence models on the accuracy of predictions for the indoor environment of poultry houses. It is, therefore, recommended that detailed studies be conducted on this topic.

In future studies, the integration of ML, BIM, and feedback control systems with CFD promises to improve the analysis of the indoor environment in poultry houses. This comprehensive approach can contribute to higher efficiency, informed decision-making, and better environmental conditions for poultry houses.

## Figures and Tables

**Table 1:** Some commercial CFD software.

Software	Company	Website (accessed on 22 May 2023)
Ansys-Fluent	Ansys, Inc. (Canonsburg, PA, USA)	https://www.ansys.com
Star-CCM+	Siemens (Plano, TX, USA)	https://plm.sw.siemens.com
Comsol-CFD	Comsol (Burlington, MA, USA)	https://www.comsol.com
Phoenics	Cham (London, UK)	https://www.cham.co.uk
Flow Simulation	Solidworks (Waltham, MA, USA)	https://www.solidworks.com
Acusolve	Altair (Troy, MI, USA)	https://www.altair.com.es
CFD++	Metacomp Technologies, Inc. (Westlake Village, CA, USA)	https://www.metacomptech.com
PAM-Flow	Pacific Engineering Systems (Sydney, NSW, Australia)	https://www.esi.com.au
Flow-3D	Flow Science, Inc. (Santa Fe, NM, USA)	https://www.flow3d.com

**Table 2:** Summary of published research on the most used k-ε turbulence models.

Reference	Turbulence Model	Validation	Remarks
Blanes-Vidal et al. [67]	Standard k-ε	Air velocity	The inclusion of a minimum of 15 indoor air velocity measurements significantly enhanced the results of CFD analysis, resulting in a significantly improved estimation of the average air velocity at bird height.
Seo et al. [61]	RNG k-ε	Air temperature, air velocity	To assess the local and overall ventilation efficiency of six broiler houses, which consisted of one conventional house and five improved houses, a comprehensive analysis was conducted utilizing CFD technology.
Bustamante et al. [65]	Standard k-ε	Air velocity	To analyze a cross-mechanical ventilation system and assess the air velocity distribution, two methodologies were employed: CFD simulations and direct measurements.
Cheng et al. [66]	Standard k-ε	Air temperature, air velocity	The application of deflectors beneath the ceiling was investigated using CFD simulations.
Tong et al. [55]	RNG k-ε	Air temperature, air velocity, and humidity	A 3D CFD model was created to simulate various parameters, including air velocity, air temperature, humidity, and heat stress indices, within a commercial layer house.
Du et al. [57]	Realizable k-ε	Air temperature, air velocity, and humidity	To ensure accuracy, a 3D CFD model was constructed based on the actual dimensions of a laying hen house. The accuracy and reliability of the model were verified by comparing the simulation outcomes with field measurements obtained at 30 distinct locations within the facility.
Cheng et al. [26]	Standard k-ε	Air temperature, air velocity	The influence of inlet position and flap configuration on airflow and temperature within a laying hen house was meticulously examined through CFD simulations.
Chen et al. [41]	Standard k-ε	Air temperature, air velocity	An investigation was conducted to explore alternative ventilation schemes to establish a suitable design for ventilation systems in cage-free hen houses.
Yang et al. [62]	RNG k-ε	Air temperature, air velocity	A novel double-duct ventilation system was introduced by merging the benefits of an exhaust air heat recovery system with a perforated duct ventilation system. This innovative approach aimed to harness the advantages of both systems for improved ventilation and energy efficiency.
Küçüktopcu et al. [36]	RNG k-ε	Air temperature, air velocity, and humidity	The CFD technique was employed to model the spatial variabilities of the microclimate within a mechanically ventilated broiler house during both summer and winter seasons.

## Data Availability

No new data were created or analyzed in this study. Data sharing is not applicable to this article.

## References

[1] Sadigov R. (2022). Rapid growth of the world population and its socioeconomic results. Sci. World J..

[2] Groher T., Heitkämper K., Umstätter C. (2020). Digital technology adoption in livestock production with a special focus on ruminant farming. Animal.

[3] Ren G., Lin T., Ying Y., Chowdhary G., Ting K. (2020). Agricultural robotics research applicable to poultry production: A review. Comput. Electron. Agric..

[4] Tunca E. (2023). Evaluating the performance of the TSEB model for sorghum evapotranspiration estimation using time series UAV imagery. Irrig. Sci..

[5] Karunathilake E., Le A.T., Heo S., Chung Y.S., Mansoor S. (2023). The path to smart farming: Innovations and opportunities in precision agriculture. Agriculture.

[6] Subeesh A., Mehta C. (2021). Automation and digitization of agriculture using artificial intelligence and internet of things. Artif. Intell. Agric..

[7] USDA Livestock and Poultry: World Markets and Trade. https://apps.fas.usda.gov/psdonline/circulars/livestock_poultry.pdf.

[8] Dos Santos R.A., da Costa J.S., Maranduba H.L., de Almeida Neto J.A., Rodrigues L.B. (2023). Reducing the environmental impacts of Brazilian chicken meat production using different waste recovery strategies. J. Environ. Manag..

[9] Jan H. The future of chicken: Poultry beyond 2050. https://www.poultryworld.net/the-industrymarkets/market-trends-analysis-the-industrymarkets-2/the-future-of-chicken-poultry-beyond-2050/.

[10] Fulton J. (2012). Genomic selection for poultry breeding. Anim. Front..

[11] Hartcher K., Lum H. (2020). Genetic selection of broilers and welfare consequences: A review. Worlds Poult. Sci. J..

[12] Wolc A. (2014). Understanding genomic selection in poultry breeding. Worlds Poult. Sci. J..

[13] Appleby M.C., Mench J.A., Hughes B.O. (2004). Poultry Behaviour and Welfare.

[14] Lindley J.A., Whitaker J.H. (1996). Agricultural Buildings and Structures.

[15] Daghir N. (2008). Broiler feeding and management in hot climates. Poultry Production in Hot Climates.

[16] Leeson S., Summers J.D. (2010). Broiler Breeder Production.

[17] Gonçalves J.C., Lopes A.M., Pereira J.L. (2023). Computational fluid dynamics modeling of ammonia concentration in a commercial broiler building. Agriculture.

[18] Oliveira C.E.A., Tinôco I.d.F.F., Sousa F.C.d., Damasceno F.A., Andrade R.R., Maciel F.d.F., Barbari M., Martins M.A. (2023). Analysis of heat and mass transfer in compost-bedded pack barns for dairy cows using computational fluid dynamics: A review. Appl. Sci..

[19] Oloyo A., Ojerinde A., Kamboh A.A. (2019). Poultry housing and management. Poultry—An Advanced Learning.

[20] Reece F., Lott B. (1982). Heat and moisture production of broiler chickens during brooding. Poult. Sci..

[21] Oloyo A. (2018). The use of housing system in the management of heat stress in poultry production in hot and humid climate: A review. Poult. Sci. J..

[22] Winn P., Godfrey E. (1967). The effect of humidity on growth and feed conversion of broiler chickens. Int. J. Biometeorol..

[23] Dozier W., Lott B., Branton S. (2005). Growth responses of male broilers subjected to increasing air velocities at high ambient temperatures and a high dew point. Poult. Sci..

[24] Hamrita T., Conway R. (2017). Effect of air velocity on deep body temperature and weight gain in the broiler chicken. J. Appl. Poult. Res..

[25] Tickle P.G., Codd J.R. (2019). Thermoregulation in rapid growing broiler chickens is compromised by constraints on radiative and convective cooling performance. J. Therm. Biol..

[26] Cheng Q., Feng H., Meng H., Zhou H. (2021). CFD study of the effect of inlet position and flap on the airflow and temperature in a laying hen house in summer. Biosyst. Eng..

[27] Lewis P. (2010). Lighting, ventilation and temperature. Br. Poult. Sci..

[28] Hoff S.J., Kandelousi M.S. (2018). HVAC Techniques for Modern Livestock and Poultry Production Systems. HVAC System.

[29] Calvet S., Cambra-López M., Blanes-Vidal V., Estellés F., Torres A. (2010). Ventilation rates in mechanically-ventilated commercial poultry buildings in Southern Europe: Measurement system development and uncertainty analysis. Biosyst. Eng..

[30] Clandinin D., Robblee A. (1954). Radiant heating: 2. Optimum floor temperatures for brooding chicks. Poult. Sci..

[31] Ghoname M., Fouda T. (2015). Improving performance of forced-air heating system in broiler house. Sci. Pap. Ser. Manag. Econom. Eng. Agric. Rural Dev..

[32] Linhoss J.E., Purswell J.L., Davis J.D. (2021). Evaluating Radiant Heater Performance Using Chick Thermal Preference and Spatial Analysis. Appl. Eng. Agric..

[33] Shaw C.T. (1992). Using Computational Fluid Dynamics.

[34] Küçüktopcu E. (2021). Use of Deterministic and Stochastic Methods in Determining Indoor Environmental Conditions of Poultry Farm. Ph.D. Thesis.

[35] Dewan A. (2011). Tackling Turbulent Flows in Engineering.

[36] Küçüktopcu E., Cemek B., Simsek H., Ni J.-Q. (2022). Computational fluid dynamics modeling of a broiler house microclimate in summer and winter. Animals.

[37] Lomax H., Pulliam T.H., Zingg D.W., Kowalewski T. (2002). Fundamentals of computational fluid dynamics. Appl. Mech. Rev..

[38] Li Y., Nielsen P.V. (2011). CFD and ventilation research. Indoor Air.

[39] Zhai Z.J., Zhang Z., Zhang W., Chen Q.Y. (2007). Evaluation of various turbulence models in predicting airflow and turbulence in enclosed environments by CFD: Part 1—Summary of prevalent turbulence models. HVAC&R Res..

[40] Kucuktopcu E., Cemek B. (2019). Evaluating the influence of turbulence models used in computational fluid dynamics for the prediction of airflows inside poultry houses. Biosyst. Eng..

[41] Chen L., Fabian-Wheeler E.E., Cimbala J.M., Hofstetter D., Patterson P. (2021). Computational fluid dynamics analysis of alternative ventilation schemes in cage-free poultry housing. Animals.

[42] Tong X., Hong S.-W., Zhao L. (2019). Using CFD simulations to develop an upward airflow displacement ventilation system for manure-belt layer houses to improve the indoor environment. Biosyst. Eng..

[43] Chen L., Fabian-Wheeler E.E., Cimbala J.M., Hofstetter D., Patterson P. (2020). Computational fluid dynamics modeling of ventilation and hen environment in cage-free egg facility. Animals.

[44] Zhang Y., Yang D., Wu R., Yang X., Li Y., Xu H. (2022). A review of research on vehicle exhaust dispersion model based on CFD simulation technology. E3S Web Conf..

[45] Stathopoulou O., Assimakopoulos V. (2008). Numerical study of the indoor environmental conditions of a large athletic hall using the CFD code PHOENICS. Environ. Model. Assess..

[46] Gaspar P.D., Barroca R.F., Pitarma R. (2003). Performance evaluation of CFD codes in building energy and environmental analysis. Build. Simul..

[47] Wang S., Zhu D. (2003). Application of CFD in retrofitting air-conditioning systems in industrial buildings. Energy Build..

[48] Nagaraj M.S., Ezhilarasan C., Kumar A.J.P., Betala R. (2018). Analysis of multipoint cutting tool temperature using FEM and CFD. Manuf. Rev..

[49] Zajaczkowski F.J., Haupt S.E., Schmehl K.J. (2011). A preliminary study of assimilating numerical weather prediction data into computational fluid dynamics models for wind prediction. J. Wind. Eng. Ind. Aerodyn..

[50] Lee D.-G., Park I.-K., Lim J.-Y. (2014). Sunroof buffeting simulation of a simplified car model using PAM-FLOW. Trans. Korean Soc. Noise Vib. Eng..

[51] Aerts J.-M., Wathes C., Berckmans D., Perry G. (2004). Environmental management for laying hens. Welfare of the Laying Hen, Proceedings of the 27th Poultry Science Symposium of the World’s Poultry Science Association (UK Branch), Bristol, UK, 17–20 July 2003.

[52] Saraz J.A.O., Rocha K.S.O., Damasceno F.A., Tinoco I.F.F., Osorio R., Tobon J.C.A. (2017). A CFD approach to assess the effects of different opening combinations in poultry houses. Rev. Bras. Eng. Agric. Ambient..

[53] Song S.-H., Lee I.-B., Hwang H.-S., Hong S.-W., Seo I.-H., Bitog J.P., Kwon K.-S., Choi J.-S. CFD Analysis and Comparison of Forced-Ventilation Systems of Poultry Houses in Corea. Proceedings of the XVIIth World Congress of the International Commission of Agricultural and Biosystems Engineering (CIGR).

[54] Zajicek M., Kic P. CFD Analysis of Broiler House Ventilation Patterns with Respect to the Poultry Welfare. Proceedings of the Rural Development.

[55] Tong X., Hong S.-W., Zhao L. (2019). CFD modelling of airflow pattern and thermal environment in a commercial manure-belt layer house with tunnel ventilation. Biosyst. Eng..

[56] Trokhaniak V., Rogovskii I., Titova L., Dziubata Z., Luzan P., Popyk P. (2020). Using CFD Simulation to Investigate the Impact of Fresh air Valves on Poultry House Aerodynamics in Case of a Side Ventilation System. Inmateh.

[57] Du L., Yang C., Dominy R., Yang L., Hu C., Du H., Li Q., Yu C., Xie L., Jiang X. (2019). Computational fluid dynamics aided investigation and optimization of a tunnel-ventilated poultry house in China. Comput. Electron. Agric..

[58] Kucuktopcu E., Cemek B. (2019). Modelling indoor environmental conditions in a commercial broiler house. J. Agric. Sci..

[59] Zou H., Yang F., Fei Y., Tang H., Zhang Y., Ye S. (2014). CFD Simulation of the Airflow in Poultry Housing with Wind Shield. Proceedings of the 8th International Symposium on Heating, Ventilation and Air Conditioning: Volume 3: Building Simulation and Information Management.

[60] Guerra Galdo E.H., Calvet Sanz S., Estellés Barber F., López Jiménez P.A. (2015). CFD model for ventilation assessment in poultry houses with different distribution of windows. Int. J. Energy Environ..

[61] Seo I.-H., Lee I.-B., Moon O.-K., Kim H.-T., Hwang H.-S., Hong S.-W., Bitog J.P., Yoo J.-I., Kwon K.-S., Kim Y.-H. (2009). Improvement of the ventilation system of a naturally ventilated broiler house in the cold season using computational simulations. Biosyst. Eng..

[62] Yang Z., Tu Y., Ma H., Yang X., Liang C. (2022). Numerical simulation of a novel double-duct ventilation system in poultry buildings under the winter condition. Build. Environ..

[63] Babadi K.A., Khorasanizadeh H., Aghaei A. (2022). CFD modeling of air flow, humidity, CO_2_ and NH_3_ distributions in a caged laying hen house with tunnel ventilation system. Comput. Electron. Agric..

[64] Wang X., Wang K. Optimizing the Pad Cooling Ventilation System of Laying Hen Barn Using CFD in Southeast China. Proceedings of the 2013 ASABE Annual Meeting.

[65] Bustamante E., García-Diego F.-J., Calvet S., Estellés F., Beltrán P., Hospitaler A., Torres A.G. (2013). Exploring ventilation efficiency in poultry buildings: The validation of computational fluid dynamics (CFD) in a cross-mechanically ventilated broiler farm. Energies.

[66] Cheng Q., Li H., Rong L., Feng X., Zhang G., Li B. (2018). Using CFD to assess the influence of ceiling deflector design on airflow distribution in hen house with tunnel ventilation. Comput. Electron. Agric..

[67] Blanes-Vidal V., Guijarro E., Balasch S., Torres A. (2008). Application of computational fluid dynamics to the prediction of airflow in a mechanically ventilated commercial poultry building. Biosyst. Eng..

[68] Jongbo A.O. (2020). Evaluation of the environmental parameters of battery-caged poultry house in the humid tropical climate. Rev. Colomb. Cienc. Anim.—RECIA.

[69] Elghardouf N., Lahlouh I., Elakkary A., Sefiani N. (2023). Towards modelling, and analysis of differential pressure and air velocity in a mechanical ventilation poultry house: Application for hot climates. Heliyon.

[70] Dağtekin M., Karaca C., Yıldız Y. (2009). Performance characteristics of a pad evaporative cooling system in a broiler house in a Mediterranean climate. Biosyst. Eng..

[71] Wang C., Cao W., Li B., Shi Z., Geng A. (2008). A fuzzy mathematical method to evaluate the suitability of an evaporative pad cooling system for poultry houses in China. Biosyst. Eng..

[72] Hui X., Zhu Q., Ji-Qin N., Li B., Shi Z., Zhao S., Wang Y. (2016). Effect of cooling pad installation on indoor airflow distribution in a tunnel-ventilated laying-hen house. Int. J. Agric. Biol. Eng..

[73] Al Assaad D.K., Orabi M.S., Ghaddar N.K., Ghali K.F., Salam D.A., Ouahrani D., Farran M.T., Habib R.R. (2021). A sustainable localised air distribution system for enhancing thermal environment and indoor air quality of poultry house for semiarid region. Biosyst. Eng..

[74] Cunha G.C.d.A., Lopes J.P., Furtado D.A., Borges V.P., Freire E.A., do Nascimento J.W. (2019). Diagnosis and validation by computational fluid dynamics of poultry house with negative pressure ventilation. Rev. Bras. Eng. Agríc. Ambient..

[75] Ma H., Tu Y., Yang X., Yang Z., Liang C. (2022). Influence of tunnel ventilation on the indoor thermal environment of a poultry building in winter. Build. Environ..

[76] Rojano F., Bournet P.-E., Hassouna M., Robin P., Kacira M., Choi C.Y. (2016). Computational modelling of thermal and humidity gradients for a naturally ventilated poultry house. Biosyst. Eng..

[77] Saraz J.A.O., Rocha K.S., Tinôco I.D.F.F., Gates R.S., Zapata O.L., Mendes L.B., Damasceno F.A. Use of CFD modeling for determination of ammonia emission in non-insulated poultry houses with natural ventilation. Proceedings of the 2011 ASABE Annual International Meeting.

[78] Knight R.M., Zhao L., Zhu H. (2021). Modelling and optimisation of a wire-plate ESP for mitigation of poultry PM emission using COMSOL. Biosyst. Eng..

[79] Pawar S.R., Cimbala J.M., Wheeler E.F., Lindberg D.V. (2010). Contaminant Dispersion within and around Poultry Houses Using Computational Fluid Dynamics.

[80] Saraz J.A.O., Tinôco I.d.F.F., Rocha K.S.O., Mendes L.B., Norton T. (2016). A CFD based approach for determination of ammonia concentration profile and flux from poultry houses with natural ventilation. Rev. Fac. Nac. Agron. Medellin.

[81] Tong X., Hong S.-W., Zhao L. (2019). CFD modeling of airflow, thermal environment, and ammonia concentration distribution in a commercial manure-belt layer house with mixed ventilation systems. Comput. Electron. Agric..

[82] Astill J., Dara R.A., Fraser E.D., Roberts B., Sharif S. (2020). Smart poultry management: Smart sensors, big data, and the internet of things. Comput. Electron. Agric..

[83] van Hooff T., Blocken B., Tominaga Y. (2017). On the accuracy of CFD simulations of cross-ventilation flows for a generic isolated building: Comparison of RANS, LES and experiments. Build. Environ..

[84] Lee I.-b., Sase S., Sung S.-h. (2007). Evaluation of CFD accuracy for the ventilation study of a naturally ventilated broiler house. Jpn. Agric. Res. Q..

[85] Li H., Rong L., Zong C., Zhang G. (2016). A numerical study on forced convective heat transfer of a chicken (model) in horizontal airflow. Biosyst. Eng..

[86] Lawson S., Woodgate M., Steijl R., Barakos G. (2012). High performance computing for challenging problems in computational fluid dynamics. Prog. Aerosp. Sci..

[87] Sadino-Riquelme M., Donoso-Bravo A., Zorrilla F., Valdebenito-Rolack E., Gómez D., Hansen F. (2023). Computational fluid dynamics (CFD) modeling applied to biological wastewater treatment systems: An overview of strategies for the kinetics integration. Chem. Eng. J..

[88] Gebremedhin K., Wu B. (2003). Characterization of flow field in a ventilated space and simulation of heat exchange between cows and their environment. J. Therm. Biol..

[89] Yeo U.-H., Lee I.-B., Kim R.-W., Lee S.-Y., Kim J.-G. (2019). Computational fluid dynamics evaluation of pig house ventilation systems for improving the internal rearing environment. Biosyst. Eng..

[90] Doumbia E.M., Janke D., Yi Q., Amon T., Kriegel M., Hempel S. (2021). CFD modelling of an animal occupied zone using an anisotropic porous medium model with velocity depended resistance parameters. Comput. Electron. Agric..

[91] Gautam K.R., Rong L., Iqbal A., Zhang G. (2021). Full-scale CFD simulation of commercial pig building and comparison with porous media approximation of animal occupied zone. Comput. Electron. Agric..

[92] Cheng Q., Wu W., Li H., Zhang G., Li B. (2018). CFD study of the influence of laying hen geometry, distribution and weight on airflow resistance. Comput. Electron. Agric..

[93] Zhang Z., Zhang W., Zhai Z.J., Chen Q.Y. (2007). Evaluation of various turbulence models in predicting airflow and turbulence in enclosed environments by CFD: Part 2—Comparison with experimental data from literature. HVAC&R Res..

[94] Tian J., Qi C., Sun Y., Yaseen Z.M., Pham B.T. (2021). Permeability prediction of porous media using a combination of computational fluid dynamics and hybrid machine learning methods. Eng. Comput..

[95] Le D.K., Yoon J.Y. (2023). A hybrid CFD–Deep learning methodology for improving the accuracy of pressure drop prediction in cyclone separators. Chem. Eng. Res. Des..

[96] Jafarizadeh A., Ahmadzadeh M., Mahmoudzadeh S., Panjepour M. (2023). A New Approach for predicting the pressure drop in various types of metal foams using a combination of CFD and machine learning regression models. Transp. Porous Media.

[97] Usman A., Rafiq M., Saeed M., Nauman A., Almqvist A., Liwicki M. Machine learning computational fluid dynamics. Proceedings of the 2021 Swedish Artificial Intelligence Society Workshop (SAIS).

[98] Du K., Chen B. (2023). A hybrid semi-supervised regression based machine learning method for predicting peak wind loads on a group of buildings. Eng. Struct..

[99] He Y., Liu X.-H., Zhang H.-L., Zheng W., Zhao F.-Y., Schnabel M.A., Mei Y. (2021). Hybrid framework for rapid evaluation of wind environment around buildings through parametric design, CFD simulation, image processing and machine learning. Sustain. Cities Soc..

[100] Lu Y., Wu Z., Chang R., Li Y. (2017). Building information modeling (BIM) for green buildings: A critical review and future directions. Autom. Constr..

[101] Yan J., Kensek K., Konis K., Noble D. (2020). CFD visualization in a virtual reality environment using building information modeling tools. Buildings.

[102] Huang Y., Zhou X., Cao B., Yang L. (2020). Computational fluid dynamics-assisted smoke control system design for solving fire uncertainty in buildings. Indoor Built Environ..

[103] Li Y., Tong Z. (2021). Development of real-time adaptive model-free extremum seeking control for CFD-simulated thermal environment. Sustain. Cities Soc..

[104] Wu W., Wang B., Malkawi A., Yoon N., Sehovic Z., Yan B. (2018). A method toward real-time CFD modeling for natural ventilation. Fluids.

[105] Zuo W., Chen Q. (2010). Fast and informative flow simulations in a building by using fast fluid dynamics model on graphics processing unit. Build. Environ..

